# Supplementary material for: HIVprotI: an integrated web based platform for prediction and design of HIV proteins inhibitors
Source: J Cheminform. 2018 Mar 9;10:12. doi: 10.1186/s13321-018-0266-y (PMC5845081; doi:10.1186/s13321-018-0266-y)
Supplement: Supplementary file 2 — Additional file 2. Source code of HIVProtI web server. [file 13321_2018_266_MOESM2_ESM.zip › HIVprotI_Source-code/hivprotI/weka-3-6-6/doc/weka/classifiers/rules/ZeroR.html]

ZeroR


---


|  |  |  |
| --- | --- | --- |
| **Overview** \| **Package** \| **Class** \| **Tree** \| **Deprecated** \| **Index** \| **Help** \| **Weka's home** | |  |
| **PREV CLASS**   NEXT CLASS | **FRAMES**    **NO FRAMES**     **All Classes** |  |
| SUMMARY: NESTED \| FIELD \| CONSTR \| METHOD | DETAIL: FIELD \| CONSTR \| METHOD |  |


---


## weka.classifiers.rules Class ZeroR

```
java.lang.Object
  weka.classifiers.Classifier
      weka.classifiers.rules.ZeroR
```

**All Implemented Interfaces:**: java.io.Serializable, java.lang.Cloneable, Sourcable, CapabilitiesHandler, OptionHandler, RevisionHandler, WeightedInstancesHandler

---

``` public class ZeroR extends Classifier implements WeightedInstancesHandler, Sourcable ```

Class for building and using a 0-R classifier. Predicts the mean (for a numeric class) or the mode (for a nominal class).


Valid options are:

```
 -D
  If set, classifier is run in debug mode and
  may output additional info to the console
```

**Version:**
:   $Revision: 5529 $

**Author:**
:   Eibe Frank (eibe@cs.waikato.ac.nz)

**See Also:**: Serialized Form

---

**Constructor Summary**

`ZeroR()`


| **Method Summary** | |
| --- | --- |
| `void` | `buildClassifier(Instances instances)`             Generates the classifier. |
| `double` | `classifyInstance(Instance instance)`             Classifies a given instance. |
| `double[]` | `distributionForInstance(Instance instance)`             Calculates the class membership probabilities for the given test instance. |
| `Capabilities` | `getCapabilities()`             Returns default capabilities of the classifier. |
| `java.lang.String` | `getRevision()`             Returns the revision string. |
| `java.lang.String` | `globalInfo()`             Returns a string describing classifier |
| `static void` | `main(java.lang.String[] argv)`             Main method for testing this class. |
| `java.lang.String` | `toSource(java.lang.String className)`             Returns a string that describes the classifier as source. |
| `java.lang.String` | `toString()`             Returns a description of the classifier. |

**Methods inherited from class weka.classifiers.Classifier**

`debugTipText, forName, getDebug, getOptions, listOptions, makeCopies, makeCopy, setDebug, setOptions`

**Methods inherited from class java.lang.Object**

`equals, getClass, hashCode, notify, notifyAll, wait, wait, wait`

**Constructor Detail**

### ZeroR

```
public ZeroR()
```


**Method Detail**

### globalInfo

```
public java.lang.String globalInfo()
```

:   Returns a string describing classifier

    :   **Returns:**: a description suitable for displaying in the explorer/experimenter gui

---


### getCapabilities

```
public Capabilities getCapabilities()
```

:   Returns default capabilities of the classifier.

    :   **Specified by:**: `getCapabilities` in interface `CapabilitiesHandler` **Overrides:**: `getCapabilities` in class `Classifier`
    :   **Returns:**: the capabilities of this classifier **See Also:**: `Capabilities`

---


### buildClassifier

```
public void buildClassifier(Instances instances)
                     throws java.lang.Exception
```

:   Generates the classifier.

    :   **Specified by:**: `buildClassifier` in class `Classifier`
    :   **Parameters:**: `instances` - set of instances serving as training data **Throws:**: `java.lang.Exception` - if the classifier has not been generated successfully

---


### classifyInstance

```
public double classifyInstance(Instance instance)
```

:   Classifies a given instance.

    :   **Overrides:**: `classifyInstance` in class `Classifier`
    :   **Parameters:**: `instance` - the instance to be classified **Returns:**: index of the predicted class

---


### distributionForInstance

```
public double[] distributionForInstance(Instance instance)
                                 throws java.lang.Exception
```

:   Calculates the class membership probabilities for the given test instance.

    :   **Overrides:**: `distributionForInstance` in class `Classifier`
    :   **Parameters:**: `instance` - the instance to be classified **Returns:**: predicted class probability distribution **Throws:**: `java.lang.Exception` - if class is numeric

---


### toSource

```
public java.lang.String toSource(java.lang.String className)
                          throws java.lang.Exception
```

:   Returns a string that describes the classifier as source. The
    classifier will be contained in a class with the given name (there may
    be auxiliary classes),
    and will contain a method with the signature:

    ```
     public static double classify(Object[] i);
    ```

    where the array `i` contains elements that are either
    Double, String, with missing values represented as null. The generated
    code is public domain and comes with no warranty.

    :   **Specified by:**: `toSource` in interface `Sourcable`
    :   **Parameters:**: `className` - the name that should be given to the source class. **Returns:**: the object source described by a string **Throws:**: `java.lang.Exception` - if the souce can't be computed

---


### toString

```
public java.lang.String toString()
```

:   Returns a description of the classifier.

    :   **Overrides:**: `toString` in class `java.lang.Object`
    :   **Returns:**: a description of the classifier as a string.

---


### getRevision

```
public java.lang.String getRevision()
```

:   Returns the revision string.

    :   **Specified by:**: `getRevision` in interface `RevisionHandler` **Overrides:**: `getRevision` in class `Classifier`
    :   **Returns:**: the revision

---


### main

```
public static void main(java.lang.String[] argv)
```

:   Main method for testing this class.

    :   **Parameters:**: `argv` - the options


---


|  |  |  |
| --- | --- | --- |
| **Overview** \| **Package** \| **Class** \| **Tree** \| **Deprecated** \| **Index** \| **Help** \| **Weka's home** | |  |
| **PREV CLASS**   NEXT CLASS | **FRAMES**    **NO FRAMES**     **All Classes** |  |
| SUMMARY: NESTED \| FIELD \| CONSTR \| METHOD | DETAIL: FIELD \| CONSTR \| METHOD |  |


---
